# Canyon Diablo lonsdaleite is a nanocomposite containing c/h stacking disordered diamond and diaphite

**DOI:** 10.1098/rsta.2022.0344

**Published:** 2023-10-30

**Authors:** Péter Németh, Laurence A. J. Garvie, Christoph G. Salzmann

**Affiliations:** ^1^ Institute for Geological and Geochemical Research, Research Centre for Astronomy and Earth Sciences, Eötvös Loránd Research Network, Budaörsi út 45, Budapest 1112, Hungary; ^2^ University of Pannonia, Research Institute of Biomolecular and Chemical Engineering, Egyetem út 10, Veszprém 8200, Hungary; ^3^ Buseck Center for Meteorite Studies, Arizona State University, Tempe, AZ 85287-6004, USA; ^4^ Department of Chemistry, University College London, 20 Gordon Street, London WC1H 0AJ, UK

**Keywords:** lonsdaleite, hexagonal diamond, nanocomposite, diaphite, structural complexity

## Abstract

In 1967, a diamond polymorph was reported from hard, diamond-like grains of the Canyon Diablo iron meteorite and named lonsdaleite. This mineral was defined and identified by powder X-ray diffraction (XRD) features that were indexed with a hexagonal unit cell. Since 1967, several natural and synthetic diamond-like materials with XRD data matching lonsdaleite have been reported and the name lonsdaleite was used interchangeably with hexagonal diamond. Its hexagonal structure was speculated to lead to physical properties superior to cubic diamond, and as such has stimulated attempts to synthesize lonsdaleite. Despite numerous reports, several recent studies have provided alternative explanations for the XRD, transmission electron microscopy and Raman data used to identify lonsdaleite. Here, we show that lonsdaleite from the Canyon Diablo diamond-like grains are a nanocomposite material dominated by subnanometre-scale cubic/hexagonal stacking disordered diamond and diaphite domains. These nanostructured elements are intimately intergrown, giving rise to structural features erroneously associated with h diamond. Our data suggest that the diffuse scattering in XRD and the hexagonal features in transmission electron microscopy images reported from various natural and laboratory-prepared samples that were previously used for lonsdaleite identification, in fact arise from cubic/hexagonal stacking disordered diamond and diaphite domains.

This article is part of the theme issue 'Exploring the length scales, timescales and chemistry of challenging materials (Part 2)'.

## 1. Introduction

Between 1967 and 1968, hexagonal (h) diamond was described from laboratory samples [1,2], from the Canyon Diablo and Goalpara meteorites [3] and as the newly named mineral lonsdaleite from the Canyon Diablo iron meteorite [4]. Bundy & Kasper [1] and Cowan *et al*. [2] reported the structure of h diamond from Debye–Scherrer patterns from diffraction lines that could be indexed with a hexagonal unit cell with *a *= 2.52 Å, *c *= 4.12 Å and space group *P*6_3_*/mmc*. X-ray reflections that matched those expected for h diamond described in [1] and [2] were used to identify this phase in hard, diamond-like grains from the Canyon Diablo iron meteorite [3]. Lonsdaleite, which is the name given to the h diamond component from hard carbon grains in the Canyon Diablo iron meteorite [4], was similarly described from X-ray diffraction (XRD) data that was ‘identical in appearance with that of the wurtzite 2H polymorph of zinc sulfide, apart from differences in the spacings appropriate to the unlike cell sizes, and were completely indexed in terms of a hexagonal cell’ [4]. Thus, these early reports are consistent with lonsdaleite, named after the pioneering crystallographer Prof. Dame Kathleen Lonsdale, as the mineral analogue of h diamond.^1^

These early reports describe h diamond and lonsdaleite based on XRD data from Laue and Debye–Scherrer photographs. However, the raw XRD images are not available and these reports, except [3], only show tables of observed *d* spacings, which suggest a good match with h diamond, but hide any complexity that may have been observed in the diffraction patterns. However, these authors also indicate that their XRD data did not match pure h diamond. Frondel & Marvin [4] annotated the XRD intensities by writing ‘visual estimate of mixture with diamond’, and mentioned in the figure caption of the photo of the grains ‘cubes consisting of diamond and lonsdaleite.’ Hannemann *et al*. [3], Bundy & Kasper [1] and Cowan *et al*. [2] listed the *d* spacings of h diamond that overlapped with c diamond. Hanneman *et al*. [3] reported the XRD study of three Canyon Diablo grains, and estimated that they contain at most 30% h diamond. Thus, even the original specimens used to define lonsdaleite were described as mixtures of cubic (c) and h diamond.

Several natural and synthetic materials with diffraction data matching lonsdaleite have subsequently been reported and the name lonsdaleite is interchangeably used with h diamond. For example, it was described from meteorites [6–10], impact structures [11–13] and terrestrial sediments [14]. Its formation has been associated with high-pressure (HP) and high-temperature (HT) shock processes and its occurrence was used as evidence of asteroidal impacts, both extraterrestrial and on Earth [3,6–10]. In addition to shock formation, lonsdaleite has been reported from ultrahigh-pressure metamorphic rocks of the Kumdykol diamond deposit [15]. Its formation was assigned to conditions near 18 GPa and 1400 K [16] and it has also been reported to form from the shock compression of graphite between 20 and 200 GPa [2,17–19]. Several studies suggest an orientational relationship between graphite, lonsdaleite and c diamond [1,20–22], and it was argued to play an important role during the martensitic graphite to c diamond transition [17,21].

Early reports and numerous subsequent ones used XRD data for the identification of h diamond. A characteristic feature of the X-ray data from both laboratory-produced and terrestrial/extraterrestrial samples interpreted as h diamond/lonsdaleite are broad diffraction features. The broadness of the XRD reflections, as well as the fact that the most intense c diamond reflections coincide with the purported h diamond reflections, complicates the analysis of the patterns. As a result, the majority of the XRD data were interpreted as physical mixtures of c and h diamond, and the significant broadening was attributed to small particle sizes [3,4,23]. Even the early reports [3,4] suggested that the particle size of Canyon Diablo h diamond was below a few tens of nanometres. Similarly, Yoshiasa *et al*. [23] used Rietveld refinements for analysing the XRD data of HP and HT compressed graphite samples and determined a small (approx. 1 nm) crystallite size of h and c diamonds.

Given the apparent nanometre scale of the diffracting domains, transmission electron microscopy (TEM) has been used to probe the structure of samples that are thought to contain lonsdaleite [24–31]. In particular, selected-area electron diffraction (SAED) patterns and fast Fourier transforms (FFTs) calculated from high-resolution TEM (HRTEM) images have been used to report h diamond (Supplementary Materials in [31]). However, analogous to the XRD patterns, the electron diffraction patterns show broadening and streaking of the diffraction spots. Concerns have been raised against the h diamond identifications because its diagnostic $h0\bar{h}l$ reflections have not been observed, and the streaking of reflections can be explained from stacking disorder [24–26,31,32]. Furthermore, several studies suggest that the hexagonally arranged *hki*0 reflections are not diagnostic for h diamond and thus should not be used for its identification [24–32].

Insight into the atomic-scale structure of the Canyon Diablo hard carbon grains is provided by aberration-corrected HRTEM [24,31]. Several recent papers demonstrate that these samples, which were originally used to identify and define lonsdaleite, are dominated by nanometre-scale structural complexity [24,27–31]. In particular, distinct nanometre-sized h diamond domains are absent. Instead, the TEM data show that the grains are composed of an intimate mixture of c diamond twins and stacking faults together with diamond–graphite nanocomposites containing sp^3^-/sp^2^-bonded structures, called diaphites [27–31]. Two distinct diaphite structures were reported based on HRTEM observations combined with density functional theory (DFT) calculations. Type 1 and type 2 diaphite structures are characterized by few-layered sp^2^-bonded graphene layers inserted between {111} surfaces of c diamond and by the coherent bonding of graphene layers with the {113} c diamond surfaces, respectively. For both types the numbers of graphitic and diamond layers are variable. Thus, understanding the structural complexity of the Canyon Diablo hard carbon requires the consideration of c and h diamond stacking and associated diaphite structures.

The HRTEM data show the dominance of stacking disorder in the Canyon Diablo hard carbon grains [24,31]. As such, it is inappropriate to model the powder XRD patterns from these grains purely as a physical mixture of nano-sized h and c diamonds. In addition, such a simplistic modelling does not capture the structural complexity revealed by TEM, including the diaphite structures. Quantitative analysis of the stacking disorder is provided by the DIFFaX analysis method [33], which calculates powder patterns of layered structures with stacking disorder. The input data are the lattice constants, atomic coordinates within the layers, symmetry relationships between the layers for the different types of stacking, peak profile parameters, thermal displacement parameters and stacking probabilities. Recently, DIFFaX was extended by implementing a least-squares refinement of the various parameters [34,35]. The new approach permits Monte Carlo-type random changes of the various parameters in order to find the best fit to the diffraction data and to enable the fitting procedure to avoid local minima. This new approach, called MCDIFFaX, was applied to powder XRD data acquired from a range of samples purported to contain h diamond [26,31,35,36] as well as ice [34], silver iodide [37] and ammonium fluoride [38]. With MCDIFFaX, the XRD data can be modelled in terms of the hexagonality *Φ*_h_ parameter, which reflects the fraction of hexagonal stacking events. Accordingly, the cubicity, *Φ*_c_, reflecting the fraction of cubic stacking, is given by 1 − *Φ*_h_. MCDIFFaX can consider up to second-order stacking probabilities, which means that independent stacking probabilities can be defined that depend on up to two previous stacking events. For example, *Φ*_ccc_ is a second-order stacking probability that reflects the probability of cubic stacking following two previous cubic stacking events. Both hexagonality and cubicity can be calculated from the higher order stacking probabilities [35]. It is informative to plot the first-order stacking probabilities, *Φ*_hc_ and *Φ*_cc_, on a ‘stackogram’, where the overall hexagonality can be read off as well as information on the tendencies for either switching or staying with the same type of stacking. The application of the MCDIFFaX method to powder XRD profiles of a range of h diamond/lonsdaleite bearing samples shows that they are dominated by stacking disordered diamond, and none display a hexagonality greater than 0.6 [35].

In this contribution, we investigate the structure of the Canyon Diablo hard carbon grains from the Buseck Center for Meteorite Studies (BCMS), Arizona State University (ASU). These grains are characterized by their extreme resistance to mechanical abrasion and chemical treatment. We refer to these grains as cotype specimens although in the strict sense only the samples investigated by Frondel & Marvin [4] should be referred to as such [39]. However, our description is justified since (i) the first reports were based on materials selected from the Canyon Diablo meteorite collection of BCMS (formerly the Center for Meteorite Studies—CMS) by founding director Prof. Carleton Moore, (ii) all the Canyon Diablo hard carbon grains have similar physical appearances and display the matching characteristic structural features used for lonsdaleite identification and (iii) neither of the original specimens used for analysis correspond to pure phase lonsdaleite. We examine the X-ray and electron diffraction features of h diamond and compare it with c/h stacking disordered diamond, graphite and diaphite. We point out the problems of the XRD peak assignments with physical mixtures of c and h diamonds, reveal the diagnostic electron diffraction data of h diamond, and emphasize that the hexagonal features on TEM images are not unique for h diamond. Microfocus synchrotron XRD combined with MCDIFFaX modelling and HRTEM images demonstrate the combination of various intergrowth types among c/h stacking disordered diamond and diaphite as well as provide an explanation for the structural complexity of the Canyon Diablo specimens and other samples previously described as lonsdaleite.

## 2. Experimental procedure

### (a) Samples, XRD measurements and analysis, calculation of ED data and TEM measurements and data processing

**Figure 1.**
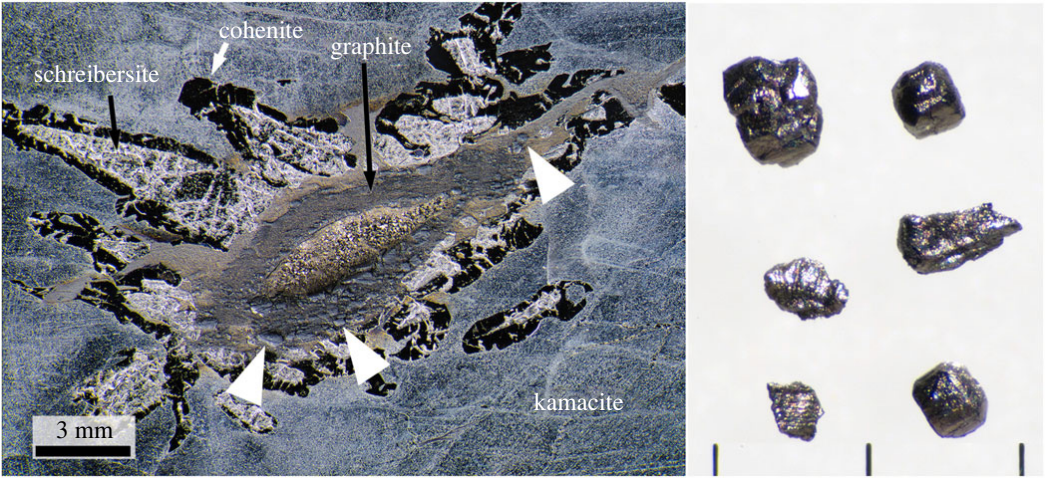
Photograph of a section of Canyon Diablo iron meteorite slice (left) containing hard carbon grains (formerly called diamonds) and a selection of grains extracted after acid dissolution and heavy density mineral separation (right). The iron was ground, polished and etched with nital and shows the characteristic mineralogy of a hard carbon-grain-bearing region. The hard carbon grains (indicated by the white arrows), which sit proud of the surface, occur within the graphite rim surrounding the crystalline troilite core. The iron meteorite slice is sample #34.102x in the Buseck Center for Meteorite Studies and is the mirror slice of the sample studied by Ksanda and Henderson in 1939 [41]. Scale bars next to the extracted hard carbon grains = 1 mm.

Hard carbon grains were extracted from the Canyon Diablo iron meteorite following the procedure described in [24,40]. The grains are black with an adamantine luster (figure 1), their sizes range from tens of micrometres to millimetres, and they showed extreme resistance to mechanical abrasion. Hard carbon grains were extracted from two shocked graphite nodules (ASU#34_140, ASU#34_141) and from a shocked graphite–troilite region in sample ASU#34_SH.

**Figure 2.**
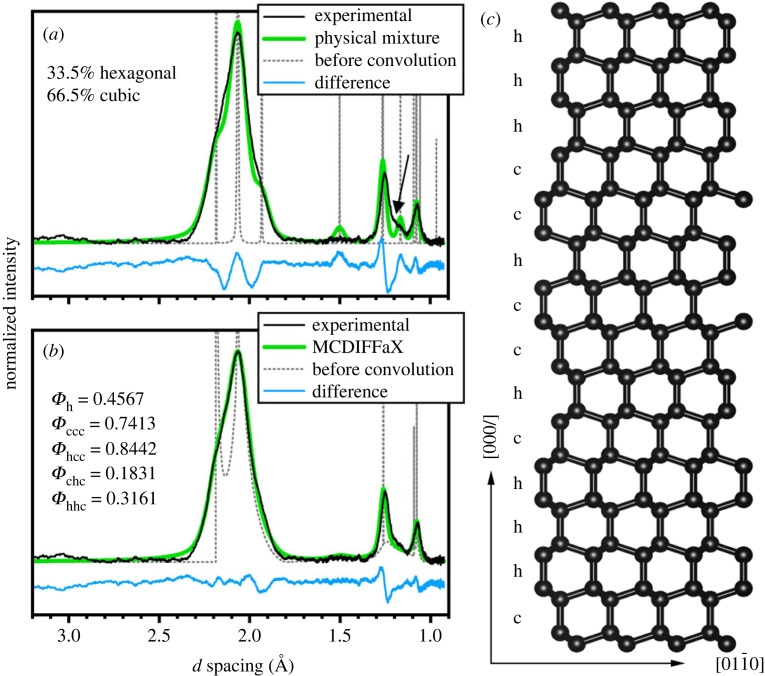
XRD data of an approximately 0.1 mm-size hard carbon grain, reported in [24], analysed (*a*) as a physical mixture of c and h diamond structures and (*b*) with c/h stacking disorder using MCDIFFaX. (*c*) Example structure of c/h stacking disordered diamond projected along $\left\langle 10\bar{1}0\right\rangle$. Hexagonal and cubic stacking are indicated by ‘h’ and ‘c’, respectively.

**Figure 3.**
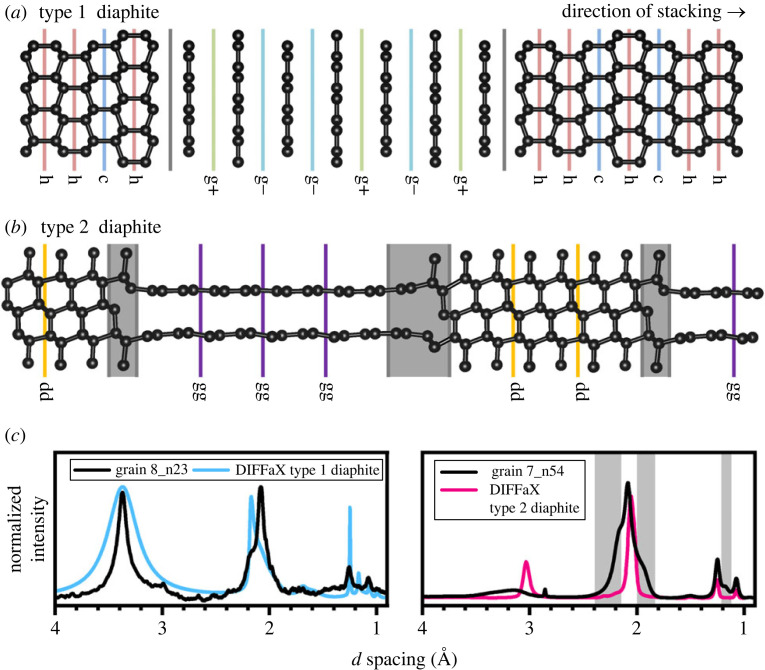
Structure model of type 1 and type 2 diaphite structures and their corresponding simulated XRD patterns plotted on XRD data measured from 2.0 × 2.0 µm^−2^ areas of two Canyon Diablo grains referred to as grain 8 and grain 7 in [31]. (*a*) For type 1 diaphite graphene layers with ‘g+’ and ‘g−’ type stackings are inserted within c/h stacking disordered diamond. (*b*) For type 2 diaphite the ‘dd’ and ‘gg’ indicate the continuations of cubic diamond and graphene regions, respectively, whereas the grey-shaded areas show the structures required to transition from cubic diamond to graphene and vice versa. (*c*) The simulated patterns of type 1 (*Φ*_c _= *Φ*_h _= 0.4, *Φ*_dg _= *Φ*_gd _= 0.2, *Φ*_g+ _= *Φ*_g− _= 0.4) and type 2 diaphite (*Φ*_gg_ = 0.9, *Φ*_dg_ = 0.0053, *Φ*_g_ = 0.1) obtained by DIFFaX analysis are shown in blue and pink, respectively, on the measured XRD patterns.

The XRD data shown in figure 2 were obtained following the measurement protocol reported in [24] from a hard carbon grain using a Bruker SMART APEX single-crystal diffractometer employing Mo*K_α_* radiation (*λ *= 0.71073 Å). The XRD datasets shown in figure 3 were obtained following the measurement protocol reported in [31] from two hard carbon grains (called grain 7 and 8) using a 2 × 2 µm X-ray beam (*λ *= 0.3738 Å) at the ID27 beamline of the ESRF-EBS synchrotron facility in Grenoble, France. Figure 3*b*,*c* correspond to the integrated profiles of the n23 and n54 two-dimensional maps from grain 8 and grain 7 shown in [31]. The data in figure 2*b*,*c* were analysed using a physical mixture of c and h diamond crystallites and the MCDIFFaX protocol based on models built for sp^3^-bonded c/h stacking disorder [35]. The diffraction patterns in figure 3*b*,*c* were investigated using the DIFFaX protocol based on models built for type 1 and type 2 diaphite structures, respectively. Details of the fitting procedure of c/h stacking disordered diamond, type 1 and type 2 diaphites were presented in [26,27,31,35,36].

Electron diffraction patterns were calculated using the Single Crystal software (CrystalMaker Software Ltd., Oxford, UK) for a thickness of 10 nm using the structures for c diamond, h diamond, c/h stacking disordered diamond and 2H graphite (figure 3). For the c/h stacking disordered diamond a CIF file of a stacking disordered structure with *Φ*_h_ = 0.5, random stacking and 120 layers was prepared with the STACKY program as described in [42]. Due to multiple scattering of electrons, only the arrangements and *d* spacings of the reflections should be compared with experimental data. The Single Crystal software does not show the 200 reflection of c diamond along $\left\langle 01\bar{1}\right\rangle$ even for 100 nm thick samples. This reflection should not occur due to the systematic absence of the d glide. However, Cowley *et al*. [43] demonstrated that this reflection has appreciable intensity for samples thicker than 5 nm based on multi-slice electron diffraction calculations and we observed it even from 2 to 4 nm thick nanodiamonds [25]. Therefore, we presume the Single Crystal software does not calculate properly the dynamically scattered electrons, and we included the 200 reflection for the c diamond calculated electron diffraction patterns.

For the TEM investigations, hard carbon grains were crushed with a pestle and mortar, suspended in distilled water and dried on lacy-C-coated Cu TEM grid. From grain 7 (reported in [31]) two lamellae, perpendicular to each other and measuring 10 × 2 µm in area and approximately 40–50 nm thickness, were cut and focus ion-beam (FIB) thinned using a Thermo Scientific Scios 2 Dual Beam equipment. The crushed grains were investigated using a JEOL JEM 4000EX (400 kV; 0.17 nm point resolution) (figure 5*a*) and an aberration-corrected JEOL ARM200F scanning (200 keV, 0.08 nm point resolution) (figures 5*b*, 6*a*,*c*, 7*a*,*b*, 8*a*,*b*, 10*a*,*c*) transmission electron microscope. The FIB lamellae were studied using a Philips CM 20 (200 keV, 0.25 nm point resolution) (SAED patterns of figure 5*c*,*d*) and an aberration-corrected Thermo Fisher Scientific FEI THEMIS 200 microscope (200 keV, 0.07 nm point resolution) (figures 5*c*,*d*, 8*d*, 9*a*, 10*d*). FFTs were calculated using Gatan Digital Micrograph v. 3.6.1 software. Note, we use four indices *hkil* (*i* = −(*h + k*)) for labelling diffraction spots, crystallographic planes and directions of h diamond, c/h stacking disordered diamond and graphite.

## 3. Results and discussion

### (a) Morphological characteristics of the hard carbon grains from Canyon Diablo

The hard carbon grains occur in shocked-transformed metal and shocked graphite nodules from the Canyon Diablo iron meteorite (figure 1). Their presence is evident during the cutting of the iron meteorites as the diamond saw blades only penetrate the regions with the hard carbon grains with extreme difficulty. These grains also protrude from the cut surfaces after grinding and polishing with SiC impregnated papers (figure 1). These hard grains are extracted by dissolving the enclosing metal as well as associated troilite with hot concentrated HNO_3_ leaving a granular black residue. Residual schreibersite was removed with a magnet. This residue is washed, dried and further separated using lithium metatungstate heavy liquid with a density of approximately 3 g cm^−3^. The high-density residue is dominated by black grains with an adamantine lustre (figure 1). Most grains are anhedral, with a crude layered structure. A small subset of the grains has a cubic morphology, presumably reflecting their formation from cliftonite, which are aggregates of graphite particles with cuboid morphology [5,44], present in the metal prior to the impact shock event on Earth. The hard carbon grains, also often called diamond, were first described from the Canyon Diablo iron meteorite in the nineteenth century [5,45], and subsequently received considerable attention, especially during the mid-twentieth century [3,4,46–49]. The meteorites with the hard carbon grains are primarily found in specimens collected from the rim of the crater [50–53], consistent with their formation from the shock wave generated by the impact of the main iron mass that formed the crater.

### (b) XRD features of c/h stacking disordered diamond

The XRD data from a single, cubic, approximately 0.1 mm hard carbon grain displays broad diffraction features including those that arise from c diamond as well as poorly resolved maxima on the ‘shoulders’ of these broad features at 2.18, 1.93 and 1.16 Å that can be indexed using the unit cell of h diamond (figure 2*a*,*b*). The relative intensities of these diffraction features match those reported for the material used for h diamond/lonsdaleite identification [2–4]. However, the 1.51 Å XRD feature, corresponding to the *d* spacing of the $\left(10\bar{1}2\right)$ h diamond, is missing. Synchrotron microbeam XRD maps obtained from 2.0 × 2.0 µm^−2^ areas of Canyon Diablo hard grains reveal their heterogeneous nanocomposite structure [31]. These patterns are characterized by diffuse and continuous rings. The high intensity of the broad rings extends across several pixels of the detector and the highest intensity features are often arranged to display a hexagonal pattern [31]. The major rings are centred at 3.34 (reduced to 3.1 Å in some areas), 2.06, 1.25 and 1.05 Å spacings, though the first ring is absent from some areas [31]. The circular integration of the wide rings from the two-dimensional dataset results in a one-dimensional pattern that shows broad XRD peaks with ‘asymmetric tails’, attributed to diffuse scattering contributions.

In stacking disordered materials, translational symmetry is maintained within the layers, yet is broken in the direction of stacking. Hence, the *l* Miller index of the Bragg peaks affected by the stacking disorder is no longer restricted to integer numbers and can take continuous values. This results in ‘streaking’ in reciprocal space that manifests itself after integration in a one-dimensional XRD pattern, as broad and asymmetric features. Figure 2*c* shows a possible structure of c/h stacking disordered diamond where cubic and hexagonal stacking is indicated by ‘c’ and ‘h’, respectively. In principle, any stacking sequence is possible including the aforementioned memory effects that define the preference for certain long-range stacking motifs. As shown in figure 2*a*,*b*, all diffraction features are broad resulting from small diffracting domains. This means that the asymmetric diffuse scattering from the stacking disorder is not easily detectable without detailed analysis. Figure 2*a* shows a standard Rietveld fit using a physical mixture of pure c and pure h diamond with variable phase fraction. The dotted grey and solid green lines show the simulated diffraction data before and after the convolution with a Pseudo-Voigt profile function, respectively. The physical mixture structural model shows significant deficiencies with respect to reproducing the experimental diffraction data shown in black. In particular, the shape of the main feature at approximately 2.1 Å is not well reproduced including the two shoulders; the intensity of the approximately 1.5 Å feature, which is absent in the experimental data, is overestimated and the asymmetric diffuse scattering at approximately 1.2 Å is not reproduced (highlighted with an arrow). Overall, this modelling illustrates that a physical mixture of pure c and pure h diamond does not adequately reproduce the experimental diffraction data.

By contrast, figure 2*b* shows an MCDIFFaX fit to the experimental diffraction allowing second-order stacking probabilities. Again, the data before the convolution with the profile function are shown in grey. In addition to sharp Bragg features, MCDIFFaX produces the diffuse asymmetric scattering arising from the stacking disorder at approximately 2.0 and 1.2 Å. After convolution with the profile function, a very good fit to the experimental data is observed. The MCDIFFaX analysis reproduces the overall shape of the main feature, does not show the occurrence of the experimentally not observed approximately 1.5 Å XRD feature and produces the diffuse asymmetric scattering at approximately 1.2 Å. The refined second-order stacking probabilities are given in figure 2*b* that result in a hexagonality of 0.4567, i.e. 45.67% hexagonal and 54.33% cubic stacking within this sample. We note that this number gives the amounts of hexagonal layer stackings, and it does not mean the sample contains 45.67% h diamonds. The corresponding first-order stacking probabilities *Φ*_cc_ and *Φ*_hc_ are 0.7655 and 0.2790, respectively. Since *Φ*_cc_ > *Φ*_c_ and *Φ*_hc_ < *Φ*_c_ means that the sample displays a preference for staying within either a cubic or hexagonal stacking sequence rather than random stacking or even preferential switching between h and c. Yet it is still sufficiently distinct from a physical mixture with essentially infinitely long cubic and hexagonal sequences. An example stacking sequence consistent with the refined second-order stacking probabilities is

hchhccchhccchhhhccchhchhchhhhhhhcchccccccchhhhhcchhhhhhcccchhhhcccccccccccchhhchcccccchhhhcccccchcccchhcchhhcchhhhhhhhhcccccccccchccchhhcccccchhhhccchhcchhhhccccccccchhhhhhhhhhcccchchhhcccccccchhhhcccccccchhhccchhccccccchhhhhccchhhhhhhhhccccchhhcchcchhhhhcccccchhhhhhcchhhhhccccchhhhccccchhcccchhhcc.

### (c) XRD features of diaphite

The structures of the two different types of diaphite are shown in figure 3*a*,*b*. For the DIFFaX models, some simplifications were made. For type 1 diaphite, the diamond regions were allowed to display c/h stacking and depending on the shifts upon stacking the graphene sheets, i.e. either g+ or g−, hexagonal and rhombohedral stacking can be achieved. Yet, no structural reconstructions were implemented at the interfaces between diamond and the stacked graphenes [31]. Type 2 was considerably more difficult to implement in DIFFaX since transition motifs between diamond and graphene regions are needed. These are shown with grey shading in figure 3*b*. As a consequence of this, it was difficult to additionally implement the c/h stacking disorder in the diamond regions. The lengths of the diamond and graphene regions are determined by the probabilities associated with the dd and gg stacking events [31].

Many XRD patterns of Canyon Diablo show an intense but broad reflection at *d* = 3.34 Å, corresponding to the interlayer spacings of graphene sheets in graphite and in type 1 diaphite (figure 3*a*). Although this broad reflection can be explained with fine-grained (1–5 nm size) graphite, this peak can also arise from type 1 diaphite since TEM investigation of the samples indicates the abundance of crystallographically intergrown graphene and c diamond layers [31]. Several of the XRD patterns display a broad maximum at 3.1 Å consistent with type 2 diaphite with ‘compressed’ interlayer spacings due to the coherent bonding between {0001} graphene layers and the {113} diamond surfaces (figure 3*b*). HRTEM data suggest the broadness of the highest *d* spacing peak can be explained by the intergrown type 1 and type 2 diaphite structures [31] as well as their variable graphene unit contents [27,28]. The major difference between the calculated XRD patterns of type 1 and type 2 diaphites is the interlayer spacings of 3.1 and 3.34 Å (figure 3*c*). The approximately 3.1 Å peak was also reported from the quenched material obtained following HP–HT treatment of graphite [54] and fullerenes [55].

Fully understanding the XRD data requires the contemporaneous consideration of c/h stacking disordered diamond combined with diaphite structures and potentially hexagonal/rhombohedral stacking disorder within the type 1 graphene regions. This represents a serious challenge. Our DIFFaX analysis is very promising with respect to dealing with this structural complexity, but the large number of parameters associated with c/h stacking disordered diamond and diaphite structures currently inhibits a full-scale refinement. The Canyon Diablo grains are heterogeneous with significant spatial variability of the sp^3^/sp^2^-bonded carbon nanostructures [31]. Certain areas are dominated by c/h stacking disordered diamond with hexagonalities up to 0.43 (figure 2*b*), whereas other areas contain a considerable amount (10–20%) of type 1 or type 2 diaphite structures (figure 3*c*).

### (d) A comparison of the electron diffraction features of h diamond with c diamond, c/h stacking disordered diamond, graphite and diaphite

The SAED and FFT patterns calculated from HRTEM images have been used as evidence for h diamond (Supplementary Material in [31]). However, this identification is problematic because the diffraction patterns along major zone axes are, within measurement error, indistinguishable from those of c diamond, c/h stacking disordered diamond, graphite and diaphite. Calibrated SAED patterns obtained using a well-aligned microscope show errors of ±1–2%, so it is challenging to distinguish between the 2.18 Å spacing $\left(10\bar{1}0\right)$ of h diamond and 2.13 Å spacing $\left(10\bar{1}0\right)$ of graphite or diaphite without an internal standard, even for well-crystallized materials. Although the 2.18 Å $\left(10\bar{1}0\right)$ of h diamond and the 2.06 Å spacing (111) of c diamond can be distinguished, the situation is further complicated for defective structures, which give rise to the broadening of the diffraction spots.

**Figure 4.**
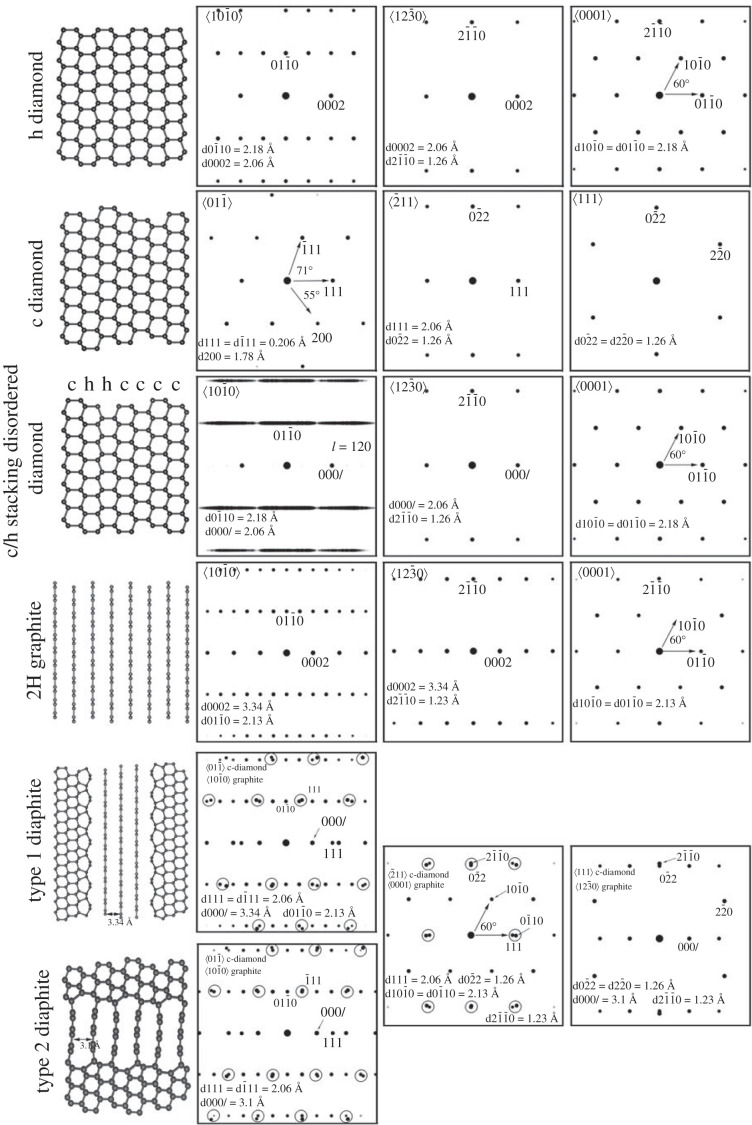
Structure models and calculated diffraction patterns of h, c and c/h stacking disordered diamond as well as 2H graphite and diaphite along major projections. The arrangement of the diffraction patterns reveals the three-dimensional crystallographic relationship, reported by Garvie *et al.* [56] and Németh *et al.* [27], among the various structures. Double reflections marked by black circles for diaphite structures are expected to occur as a broad reflection.

To further illustrate the measurement challenges, we show the calculated SAED patterns for h diamond, c diamond, c/h stacking disordered diamond, 2H graphite and type 1 and type 2 diaphites along major zone axes (figure 4). These structures are those reported from samples associated with h diamond [24–31]. A sufficiently large unit cell containing 120 layers with 50% cubic and 50% hexagonal stacking in a random stacking sequence was used for calculating diffraction patterns of c/h stacking disordered diamond. Since structural models for extended type 1 and type 2 diaphite structures are not available, their diffraction patterns were generated by adding the corresponding patterns of 2H graphite with the appropriate layer thickness, i.e. 3.34 and 3.10 Å, of type 1 and type 2 diaphites and c diamond. The reflections of c diamond and graphite are close to each other and expected to become one broad reflection for diaphites. In figure 4, the various structures and their calculated diffraction patterns are arranged according to the orientational relationships described in [27,54].

The identification of h diamond based on diffraction data is unambiguous for the $\left\langle 10\bar{1}0\right\rangle$ projection. Along this projection the h diamond reflections can be distinguished from those of c diamond, c/h stacking disordered diamond, graphite and diaphite (figure 4). The calculated electron diffraction pattern shows the distribution and the *d* spacings of the orthogonally arranged 0002 (2.06 Å) and $10\bar{1}0$ (2.18 Å) reflections of h diamond, which are distinct from any projections of the other structures. However, discrete $h0\bar{h}l$ reflections for h diamond have not been reported from any natural or synthetic material. The corresponding projection for c diamond is ⟨011⟩ with 2.06 Å *d* spacings for 111 and $\bar{1}11$ reflections and 71° between the 111_g_ and $\bar{1}11_{\text{g}}$ vectors. The c/h stacking disordered diamond along $\left\langle 10\bar{1}0\right\rangle$ can be recognized by the streaking of $01\bar{1}l$ reflections. 2H graphite and type 1 and type 2 diaphites along $\left\langle 10\bar{1}0\right\rangle$ graphite projection can be distinguished from h diamond by the occurrence of the 3.34 Å or the 3.10 Å *d* spacing of 000*l* reflections of the graphite units.

The diffraction pattern of h diamond in the $\left\langle 12\bar{3}0\right\rangle$ orientation, perpendicular to $\left\langle 10\bar{1}0\right\rangle$ projection, is indistinguishable from the crystallographically corresponding $\left\langle \bar{2}11\right\rangle$ diamond and $\left\langle 12\bar{3}0\right\rangle$ c/h stacking disordered diamond, and it is distinct from graphite and diaphite (figure 3). Since type 1 and type 2 diaphites are laterally intergrown, their projections other than $\left\langle 10\bar{1}0\right\rangle$ are identical. Similarly, the *d* spacings and the distributions of the hexagonally arranged reflections of h diamond projected along ⟨0001⟩ are identical to c/h stacking disordered diamond projected along ⟨0001⟩. Furthermore, similar patterns occur for ⟨0001⟩ graphite and diaphite, and the error of *d* value measurement makes the hexagonally arranged $\left\langle 10\bar{1}0\right\rangle$ graphite (2.13 Å) and diaphite (2.13 Å) reflections indistinguishable from that (2.18 Å) of h diamond (figure 4). Thus, h diamond identification based on the ⟨0001⟩ projection is problematic.

Unambiguous h diamond identification requires tilting of a sample so as to produce discrete $h0\bar{h}l$ reflections on diffraction patterns. However, such patterns have to date not been reported. In addition, the occurrence of hexagonally arranged reflections associated with ⟨0001⟩ projections are not unique for h diamond even for sp^3^-bonded structures.

### (e) Domain structure and various intergrowths among c/h stacking disordered diamond, graphite and diaphite

**Figure 5.**
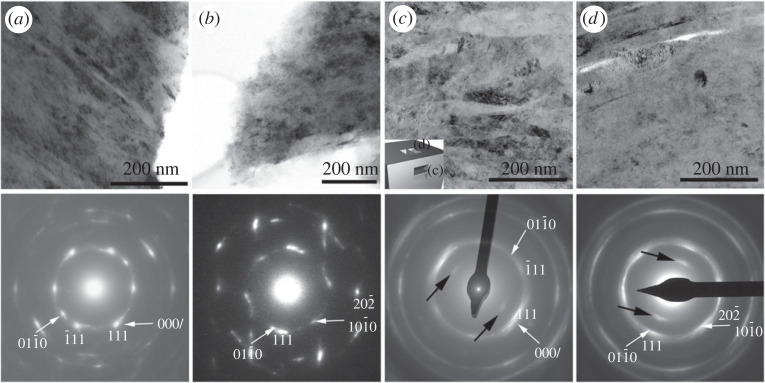
Low-magnification TEM images and corresponding SAED patterns taken from crushed grains (*a*,*b*) and FIB lamellae (*c*,*d*) of Canyon Diablo hard grains. Small inset in (*c*) shows the orientation of the FIB samples. The reflections spread over a large area roughly correspond to *d* spacings of 2.2 and 1.8 Å. The SAED patterns are indexed as $\left\langle 01\bar{1}\right\rangle$ (*a*,*c*) and $\left\langle \bar{2}11\right\rangle$ (*b*,*d*) c diamond. White arrows mark indices for $\left\langle 10\bar{1}0\right\rangle$ (*a*,*c*) and ⟨0001⟩ (*b*,*d*) c/h stacking disordered diamond. Vertical streaks on the SAED pattern (*b*) indicate (000*l*) c/h stacking disorder, i.e. the imaged area contains $\left\langle 10\bar{1}0\right\rangle$ and ⟨0001⟩ projected c/h stacking disordered diamond domains. Black arrows in (*c*) and (*d*) point to graphite 000l reflections. Original BFTEM images and SAED patterns of (*c*) and (*d*) were reported in [31] (copyright, PNAS).

Low-magnification TEM images of the Canyon Diablo specimens show 1–20 nm wide, and 100–300 nm long domains characterized by a sawtooth appearance and mottled texture (figure 5) [24,31]. The corresponding SAED patterns show spotty rings with streaking and smeared intensities. The reflections spread over a large area roughly corresponding to *d* spacings of 2.2 and 1.8 Å. The strongest reflections from some SAED patterns can be assigned to $\left\langle 01\bar{1}\right\rangle$ c diamond (figure 5*a*), though such an assignment only approximates the complexity of the patterns. The SAED pattern of figure 5*a* shows streaking of reflections from multiple $\left\langle 10\bar{1}0\right\rangle$ c/h stacking disordered diamond domains. Other SAED patterns show approximately hexagonally arranged reflections with approximately 2.1 Å *d* spacings (figure 5*b*), which are consistent with ⟨0001⟩ c/h stacking disordered diamond as well as with type 2 diaphite intergrowth consisting of $\left\langle \bar{2}11\right\rangle$ c diamond and ⟨0001⟩ graphene units.

The structure of a hard carbon grain was investigated from two FIB thinned lamellae, which were prepared from two areas after rotating the grain by 90° (figure 5*c*). The low-magnification TEM image of lamella 1 reveals a feathery texture (figure 5*c*), which may reflect previous graphitic layering arranged into bundles [31]. The corresponding SAED pattern, taken from an approximately 200 nm size area, is characterized by quasi-continuous rings, of which the strongest intensity can be indexed as $\left\langle 01\bar{1}\right\rangle$ c diamond (figure 5*c*). However, similar to figure 5*a*, the pattern displays streaking of reflections from multiple $\left\langle 10\bar{1}0\right\rangle$ c/h stacking disordered diamond domains, and their associations with graphene layers are consistent with type 1 diaphite. The feathery texture is absent on the low-magnification TEM image of lamella 2 (figure 5*d*). The corresponding SAED pattern shows continuous rings and the intensity distribution around the 111 diamond spots reveals a quasi-sixfold symmetry, which can be explained with ⟨0001⟩ c/h stacking disordered diamond as well as with type 2 diaphite intergrowth.

**Figure 6.**
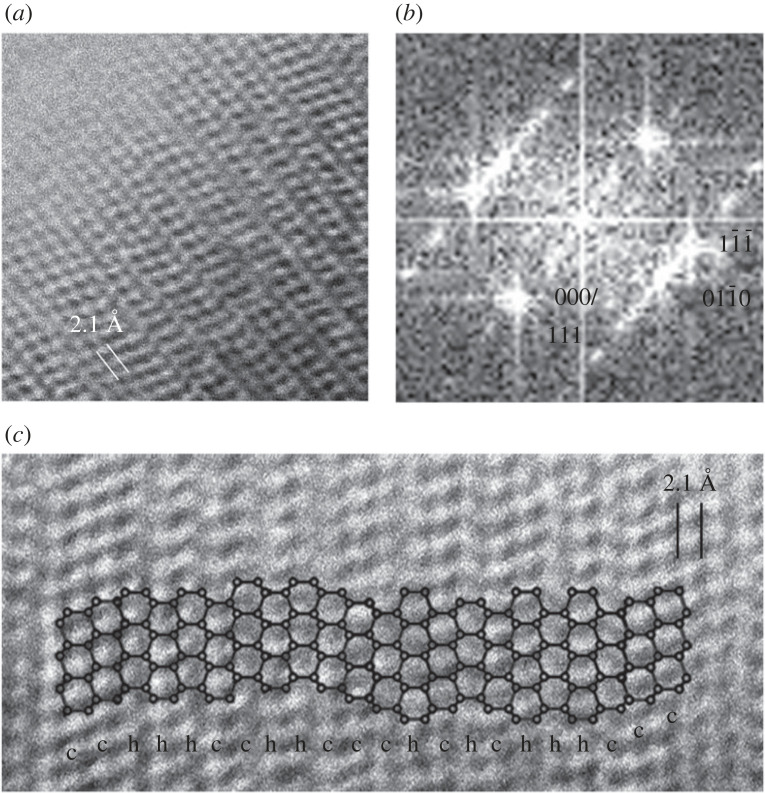
HRTEM image of c/h stacking disordered diamond (*a*), with corresponding FFT indexed as c diamond and c/h stacking disordered diamond (*b*), and its structure model plotted on an HRTEM image (*c*). Original HRTEM image (*a*) was reported in [24], copyright Springer Nature Ltd.

Understanding the complexity of the samples requires the use of HRTEM images, which reveal abundant (111) c diamond stacking faults and twins along $\left\langle 01\bar{1}\right\rangle$, i.e. they provide evidence for $\left\langle 10\bar{1}0\right\rangle$ c/h stacking disordered diamond domains (figure 6). These domains are 1–5 nm wide [24] and they are evidenced by the sawtooth appearance on low-magnification TEM images (figure 5). The structures correspond to that of c/h stacking disordered diamond, which gives rise to the streaking and the *d* spacings previously associated with h diamond. Although discrete c diamond reflections along $\left\langle 01\bar{1}\right\rangle$ can be recognized, the overall diffraction features of the FFTs (figure 6*b*) are similar to the calculated patterns based on $\left\langle 10\bar{1}0\right\rangle$ c–h stacking disordered diamond structure (figure 4).

**Figure 7.**
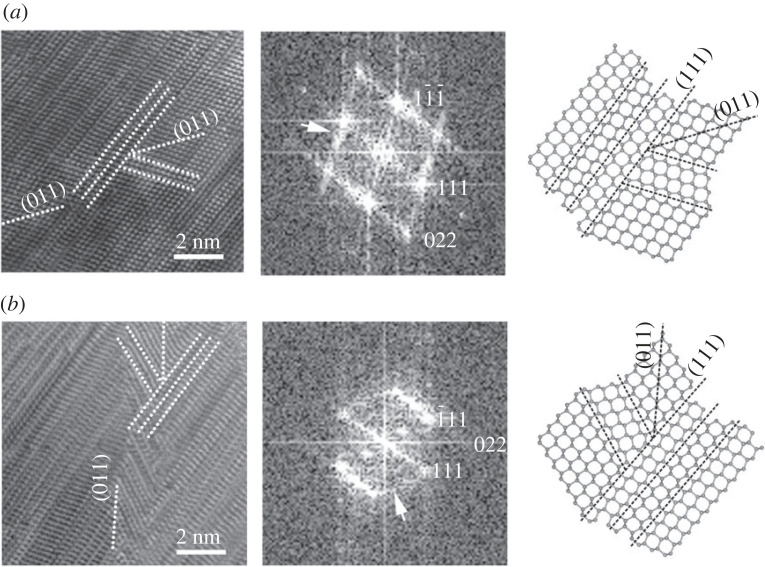
Complex (011) twins (*a*,*b*) with corresponding FFTs, and their structure models. The indices refer to c diamond structure. White arrows on the FFTs (*a*,*b*) mark a second set of streaking that resulted from the (011) twins. Original HRTEM images (*a*) were reported in [24], copyright Springer Nature Ltd.

Németh *et al*. [24] reported that multiple (111) and $\left(\bar{1}11\right)$ twins and stacking faults prevent the cubic symmetry of diamond for localized nanometre-sized regions and result in complex (011) twin intergrowths (figure 7*a*,*b*). Since the corresponding indices for c/h stacking disordered diamond are non-integer numbers, we report c diamond indices of this twin only. Although Németh *et al*. [24] reported {113} c diamond twin intergrowth also, the reinvestigation implies the reported structure is more consistent with (011) c diamond type (figure 7*b*). This twin gives rise to a second set of streaking at 71° with respect to the strong {111} c diamond or the {000*l*} c/h stacking disordered diamond streaking in FFT, which gives rise to the characteristic ring SAED patterns (figure 7*a*,*b*) and provides an explanation for the prominent sawtooth appearance of the low magnification TEM images (figure 5).

**Figure 8.**
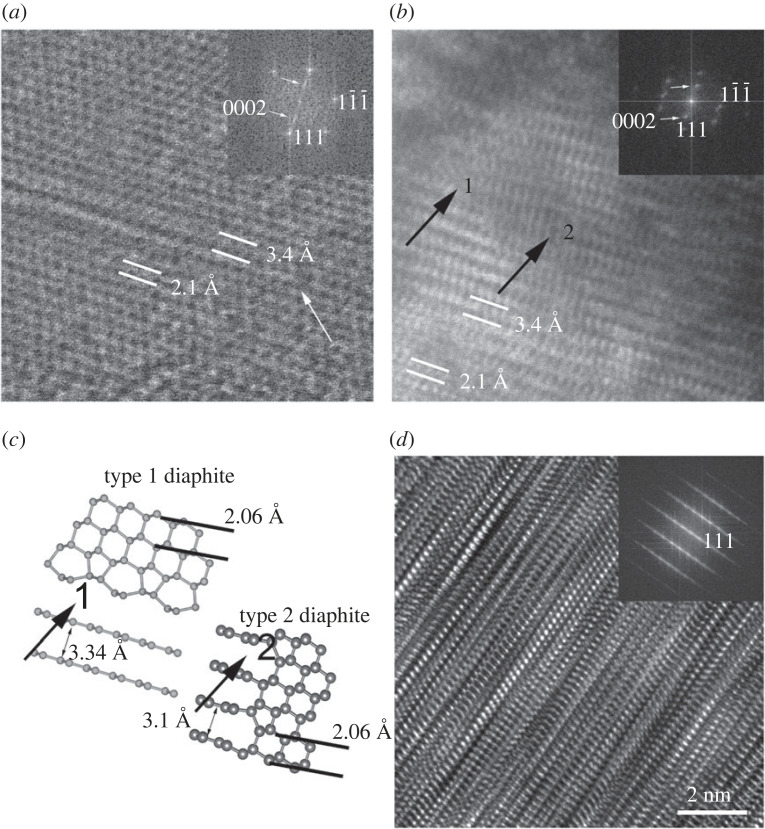
HRTEM images of type 1 and type 2 diaphite structures viewed along $\left\langle 01\bar{1}\right\rangle$ c diamond and their corresponding FFTs. Type 1 and type 2 diaphite intergrowth (*a*,*b*) and their corresponding structure model (*c*). Vertically superimposed diaphite and c/h stacking disordered diamond structures give rise to complexity for an approximately 50–60 nm thick sample (*d*).

The occurrence of diaphite structures within c and c/h stacking disordered diamond gives rise to additional complexity (figure 8). These nanostructures display lattice fringe spacings of 3.4 Å, corresponding to the graphene interlayer spacings, and 2.1 Å, consistent with both $10\bar{1}0$ graphite and 111 diamond reflections [27–31]. The 3.4 Å fringes occur systematically as few-layered graphene to graphitic domains and contiguous with the {111} diamond layers (figure 8). Their lateral extent varies up to a few nanometres and is consistent with type 1 diaphite. These structures terminate within the sp^3^-bonded lattice and give rise to type 2 diaphite (figure 8*a*,*b*). DFT calculations suggest the interlayer spacings of type 2 diaphite are compressed (3.1 Å) by the necessity of coherent bonding between the edges of the graphene layers and the {113} diamond surfaces (figure 8*c*). However, we should note that this 3.1 Å spacing cannot be reliably measured from the experimental HRTEM images.

HRTEM images show the diversity of type 1 and type 2 diaphite, and their various intergrowth structures within the grains (figure 8). Their presence is clearest in thin (less than 20 nm) samples (figure 8*a*,*b*) and is challenging to identify in samples thicker than approximately 20 nm due to the vertically superimposed nanodomains. However, the contributions of $\left\langle 10\bar{1}0\right\rangle$ graphene and the $\left\langle 01\bar{1}\right\rangle$ c diamond units can be detected in FFTs. The thicker the sample, the more complex the image. An interesting example of complexity is shown in figure 8*d*, which displays vertically superimposed diaphites structures with crystallographically intergrown c/h stacking disordered diamond.

### (f) Explanation of the hexagonally arranged reflections

Discrete, hexagonal reflections with approximately 2.1 Å spacings were previously used as evidence for h diamond. These diffraction patterns are commonly shown together with electron-energy loss spectroscopy (EELS) data. Based on the lack of obvious spectral features below 288 eV (the main onset of the diamond peak) in C K-edge core-loss EELS data, it has been claimed that samples did not contain sp^2^-bonded structures and the hexagonal reflections were assigned to h diamond (Supplementary Material in [31])). However, this assignment raises issues because (i) a reference h diamond EELS is unavailable and the reported data cannot be distinguished from c/h stacking disordered diamond and (ii) the diffraction features are not unique for h diamond even when considering only sp^3^-bonded structures.

Our calculated diffraction patterns demonstrate that c/h stacking disordered diamond is indistinguishable from h diamond along ⟨0001⟩ (figure 4). The contribution of the ⟨111⟩ c diamond stacking is hidden within ⟨0001⟩ hexagonally stacked diamond, and the $0\bar{2}2$ c diamond reflections having 1.26 Å *d* spacing overlap with those of $2\bar{1}\bar{1}0$ c/h stacking disordered diamond (1.26 Å). To identify the various stackings, 90° rotation would be necessary, which is not possible within a transmission electron microscope. However, there is abundant evidence for c/h stacking disordered diamond projected along $\left\langle 10\bar{1}0\right\rangle$ (figures 5–8), thus we suggest a portion of the hexagonally arranged reflections is explained by this structure.

**Figure 9.**
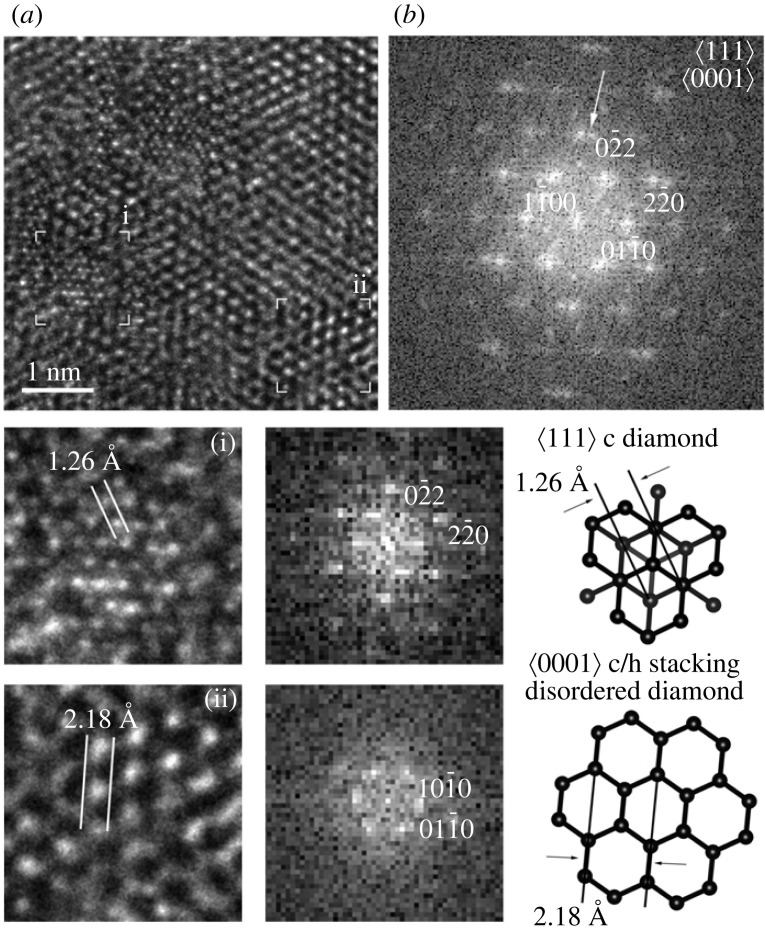
uHRTEM image (*a*) and its corresponding FFT (*b*) show evidence for ⟨111⟩ c diamond and ⟨0001⟩ c/h stacking disordered diamond intergrowth. White arrow marks in (*b*) split reflections with an approximately 10° rotation.

Ultra-high-resolution TEM (uHRTEM) images provided by aberration-corrected microscopes are necessary to resolve interatomic spacings below 1.3 Å, i.e. the 1.26 and 1.09 Å spacings corresponding to {220} and {113} diamond fringes. In a conventional (non-aberration corrected) HRTEM, only the 2.1 Å sets of {111} diamond fringes are visible. The uHRTEM image of figure 9*a* shows an intricate lattice fringe image. Its high resolution and the approximately 10° rotation between the cubic and the hexagonal stackings provide evidence for a unique c diamond and c/h stacking disordered diamond intergrowth. In certain areas of the image the 1.26 Å *d* spacing cross fringes (i) occur together with those of approximately 2.1 Å (ii). The FFT (figure 9*b*) of the image reveals split reflections having 1.26 Å *d* spacings with an approximately 10° rotation. We associate the FFT-calculated diffraction data from area i and ii with ⟨111⟩ c diamond and ⟨0001⟩ c/h stacking disordered diamond, respectively.

**Figure 10.**
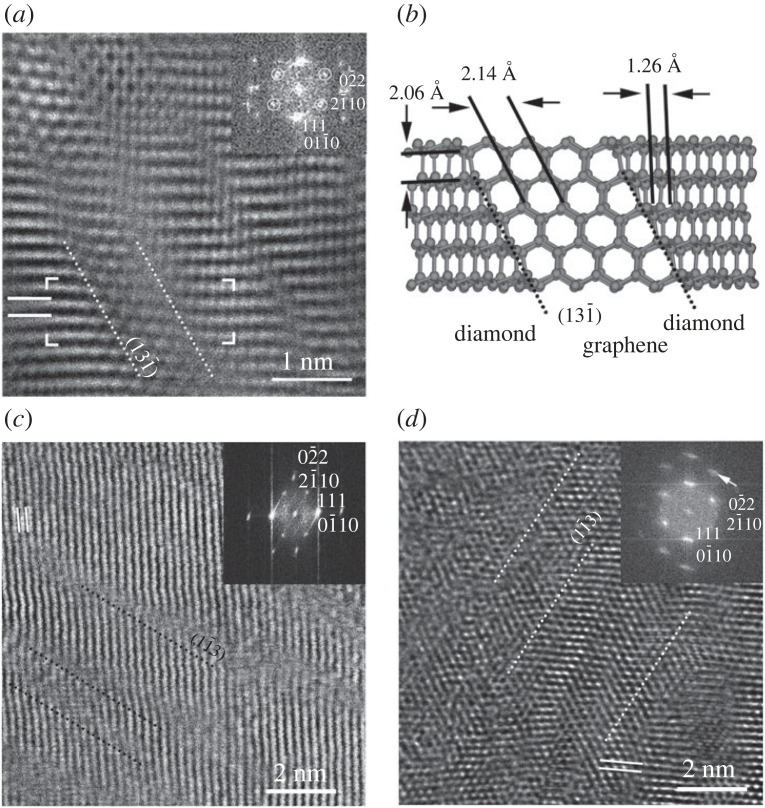
Type 2 diaphite viewed along $\left\langle 12\bar{1}\right\rangle$ c diamond and ⟨0001⟩ graphene. The original HRTEM image of (*a*) and the structure model (*b*) are reported in [24] (copyright Springer Nature Ltd.) and [27] (copyright ACS). White circles in (*a*) mark hexagonally arranged reflections and the dotted lines indicate the interface between $\left(13\bar{1}\right)$ c diamond and (0001) graphene. Type 2 diaphite examples with variable c diamond and graphene contents (*c*). Black dotted lines mark the interface between $\left(1\bar{1}3\right)$ c diamond and (0001) graphene. The $\left\langle 12\bar{1}\right\rangle$ c diamond contribution is evidenced by the $\left(1\bar{1}3\right)$ elongation of the domains (white dotted lines) and the streaking of reflections is marked by a white arrow (*d*) in a 50–70 nm thick sample.

Similar to ⟨0001⟩ c/h stacking disordered diamond, type 2 diaphite structures give rise to hexagonal reflections with 2.1 Å *d* spacings (figure 10). We note that the superimposed c diamond and graphene units of type 1 diaphite would also result in a similar pattern (figure 4). Type 2 diaphite projected along $\left\langle \bar{2}11\right\rangle$ c diamond exhibits hexagonally arranged graphene layers inserted within and bonded at high angles to the sp^3^-bonded $\left(13\bar{1}\right)$ c diamond surfaces (figure 10). HRTEM images of type 2 diaphite from the Canyon Diablo hard carbon grains (figure 10) are characterized by elongated $\left\langle 12\bar{1}\right\rangle$ c diamond domains parallel to $\left(1\bar{3}1\right)$ and subnanometre-sized regions containing approximately 2.1 Å fringes arranged in a hexagonal pattern. Németh *et al*. [24] interpreted figure 10*a* as two- and four-layer thick {113} c diamond twins. However, the DFT-based structure model (figure 10*b*) shows that such images are in fact consistent with type 2 diaphite and indicate that these nanostructures correspond to a nanocomposite material consisting of sp^2^- and sp^3^-bonded carbon regions. Type 2 diaphite contains c diamond, graphene and interface regions. By changing the volume of these regions, various type 2 diaphite structures can be generated and identified in HRTEM images (figure 10*c*,*d*). Several HRTEM images are dominated by c diamond and interface regions, and they are presumably vertically embedded within c diamond. It is an interesting question how their electronic structure and EELS data differ from c or c/h stacking disordered diamond. As the sample gets thicker, the identification of the c diamond units becomes challenging (figure 10*d*). However, the $\left(1\bar{1}3\right)$ elongation of the domains and the streaking of reflections are indicative of type 2 diaphite (figure 10*d*).

## 4. What is lonsdaleite?

Lonsdaleite is the mineral name given to the h diamond component in the hard carbon grains from the Canyon Diablo iron meteorite [4]. Since its first description in 1967, natural and synthetic materials with diffraction data matching the Canyon Diablo lonsdaleite have been widely reported. For example, from meteorites [6–10,57], impact structures [11–13] and terrestrial sediments [14]. Structural features used for lonsdaleite identification are known from diamonds occurring in various terrestrial geological settings including ultrahigh-pressure metamorphic rocks [15] and cratonic lamproite colluviums [58], in highly strained, mechanically twinned pink diamonds [59] as well as in nanodiamonds found in primitive meteorites [25,29] and synthetic materials unrelated to shock [2,20,22,23]. While lonsdaleite and h diamond have been used as synonyms, it is necessary to distinguish between the two and use h diamond for the fully hexagonal h diamond polytype exclusively. Despite these numerous reports, our data show that the XRD reflections originally identified as lonsdaleite from the Canyon Diablo hard carbon grains arise from materials dominated by highly disordered c/h stacking combined with crystallographically intergrown diaphite, and not from a discrete h diamond component. This structural complexity gives rise to intricate HRTEM images and continuous streaking of reflections on diffraction patterns, consistent with the absence of three-dimensional repetitions, even on the nanometre scale. In conclusion, Canyon Diablo lonsdaleite is not a discrete phase, but instead is a nanocomposite of disordered c/h stacked diamond and diaphite.

## Data Availability

This article has no additional data.

## Footnotes

1 The black hard carbon grains found in some specimens of the Canyon Diablo iron meteorite have been described as ‘diamonds’ since their discovery in 1891 [5]. Subsequent work has shown these grains to have a complex structure and not pure cubic diamond. As such, in this paper we refer to these grains as ‘Canyon Diablo hard carbon grains’.
